# Sexual Aggression Victimization and Perpetration among Male and Female College Students in Chile

**DOI:** 10.3389/fpsyg.2016.01354

**Published:** 2016-09-21

**Authors:** Isabell Schuster, Barbara Krahé, Paola Ilabaca Baeza, José A. Muñoz-Reyes

**Affiliations:** ^1^Department of Psychology, University of PotsdamPotsdam, Germany; ^2^Escuela de Psicología - Centro Cielo, Universidad Santo TomásSantiago, Chile; ^3^Centro de Estudios Avanzados, Universidad de Playa AnchaValparaíso, Chile

**Keywords:** sexual aggression, victimization, perpetration, prevalence, coercive strategies, relationship constellations, alcohol, Chile

## Abstract

Evidence on the prevalence of sexual aggression among college students is primarily based on studies from Western countries. In Chile, a South American country strongly influenced by the Catholic Church, little research on sexual aggression among college students is available. Therefore, the purpose of the present study was to examine the prevalence of sexual aggression victimization and perpetration since the age of 14 (the legal age of consent) in a sample of male and female students aged between 18 and 29 years from five Chilean universities (*N* = 1135), to consider possible gender differences, and to study the extent to which alcohol was involved in the reported incidents of perpetration and victimization. Sexual aggression victimization and perpetration was measured with a Chilean Spanish version of the Sexual Aggression and Victimization Scale (SAV-S), which includes three coercive strategies (use or threat of physical force, exploitation of an incapacitated state, and verbal pressure), three victim-perpetrator constellations (current or former partners, friends/acquaintances, and strangers), and four sexual acts (sexual touch, attempted sexual intercourse, completed sexual intercourse, and other sexual acts, such as oral sex). Overall, 51.9% of women and 48.0% of men reported at least one incident of sexual victimization, and 26.8% of men and 16.5% of women reported at least one incident of sexual aggression perpetration since the age of 14. For victimization, only few gender differences were found, but significantly more men than women reported sexual aggression perpetration. A large proportion of perpetrators also reported victimization experiences. Regarding victim-perpetrator relationship, sexual aggression victimization and perpetration were more common between persons who knew each other than between strangers. Alcohol use by the perpetrator, victim, or both was involved in many incidents of sexual aggression victimization and perpetration, particularly among strangers. The present data are the first to provide a systematic and detailed picture of sexual aggression among college students in Chile, including victimization and perpetration reports by both men and women and confirming the critical role of alcohol established in past research from Western countries.

## Introduction

Being forced to engage in unwanted sexual activities is a serious violation of the right to sexual self-determination. It is a major problem worldwide (WHO, 2013), associated with a wide range of negative effects on survivors' wellbeing (Martin et al., 2011; Choudhary et al., 2012). Studies demonstrating the high prevalence of sexual aggression as reported by victims and perpetrators have been conducted primarily in Western countries (Fisher et al., 2010; Krahé et al., 2014, for reviews). By comparison, little research is available on the problem of sexual aggression in developing countries. In Chile, one of the South American countries with the highest human development index (United Nations Development Programme, 2015), research on sexual aggression victimization and perpetration is notably limited. To address this gap, the primary purpose of the present study was to investigate the prevalence of experiencing and engaging in sexual aggression in a sample of female and male college students in Chile. Additionally, the present study examined the role of alcohol in sexual aggression victimization and perpetration. We defined sexual aggression as *behavior carried out with the intent or result of making another person engage in sexual activity despite his or her unwillingness to do so* (Krahé et al., 2015), considering different coercive strategies, victim-perpetrator relationship constellations, and sexual acts.

Since Koss et al.'s (1987) groundbreaking research on sexual aggression among college students in the 1980s, many large-scale studies, primarily from the United States, revealed high prevalence rates of sexual aggression. For instance, in the Campus Climate Survey on Sexual Assault and Sexual Misconduct that included 27 higher education institutions in the United States, 18.1% of female students and 4.2% of male students reported the experience of completed penetration or sexual touch through the use of physical force or incapacitation since entering college (Cantor et al., 2015). In line with this finding, a large body of research has shown higher victimization rates for women than for men (e.g., Black et al., 2011; Williams et al., 2014). At the same time, evidence is accumulating that men also experience sexual victimization to a substantial degree (e.g., French et al., 2015; Krahé et al., 2015).

Fewer studies have studied the prevalence of reported perpetration of sexually aggressive behavior, especially in community or college student samples. Recent studies from the U.S. found that about 30% of male college students reported some form of sexually aggressive behavior since adolescence (Swartout et al., 2015; Dardis et al., 2016). In a recent German study, the overall prevalence of male perpetration was lower, but still substantial with 13.2% (Krahé and Berger, 2013). By comparison, perpetration rates are generally lower for women than for men (e.g., Struckman-Johnson, 1988; D'Abreu et al., 2013; Tomaszewska and Krahé, 2015), but it is clear that sexually aggressive behavior is not limited to males. Comparing prevalence rates of both sexual aggression perpetration and victimization, many studies found a substantial gap, with higher prevalence rates for sexual victimization (Kolivas and Gross, 2007, for a review).

Although the majority of large-scale studies focused on female victims and male perpetrators (e.g., WHO, 2013; Abrahams et al., 2014), there is evidence to show that men may also be victims of sexual aggression (Peterson et al., 2011, for a review) and women may also be perpetrators (Fisher and Pina, 2013, for a review). In a cross-cultural study conducted in 10 European countries, college men and women reported sexual aggression victimization and perpetration since the legal age of consent in the respective country (Krahé et al., 2015). The overall rates for sexual victimization were 32.2% for women and 27.1% for men. With respect to the perpetration of sexual aggression, the overall rates were 16.3% for men and 5.0% for women across all countries. These results challenge traditional stereotypes about sexual aggression with women as victims and men as perpetrators. Therefore, a gender-inclusive approach to sexual aggression is necessary to capture all facets of sexual aggression and provide support to both female and male victims (Turchik et al., 2016).

Regarding the relationship constellation between victim and perpetrator, studies found consistently that sexual aggression more often involves victims and perpetrators known to each other than happening between complete strangers (Black et al., 2011; Lehrer et al., 2013a). With respect to situational facilitators of sexual aggression, alcohol has been found to be involved in about half of all incidents of sexual assault (see Abbey et al., 2004, for a review), especially when victim and perpetrator are not well-acquainted (Ullman et al., 1999; Abbey et al., 2003). Alcohol increases the risk of both perpetration and victimization through its pharmacological effects by impairing information processing, such as attention to norms prohibiting the use of coercion or attention to danger signals. Alcohol also increases the risk of sexual aggression through its psychological effects, such as the activation of stereotyped beliefs about drinking alcohol as a cue indicating sexual availability and the risk of men overestimating women's sexual interest (Abbey et al., 2004).

### Sexual aggression among college students in chile

As noted above, there is a large body of research on sexual aggression in Western countries, highlighting its prevalence and costs for individuals and society. By contrast, data from Chile on sexual aggression victimization and perpetration, particularly among young adults, is limited. In Chile, the Catholic Church is a strong source of influence on social norms and public policy, in particular referring to sexuality (Lehrer et al., 2009; Morán Faúndes, 2013). For example, abortion is illegal under any circumstances, including incest or rape (Amnesty International, 2015), and divorce, which was not legalized until 2004, is opposed by the Catholic Church (Blofield, 2006). Despite this strong influence of the Catholic Church, norms and sexual life style of young adults have been changing over the last decades, which is reflected, for instance, in a younger age at sexual initiation (INJUV, 2013) and a higher number of sexual partners among women (González et al., 2007). These changes in sexual lifestyle make the study of sexual aggression perpetration and victimization in Chile even more critical.

To date, there are three published data sets on sexual assault victimization among college students in Chile (Maida et al., 2003; Lehrer et al., 2013a; Ilabaca et al., 2015), which produced similar prevalence rates. In a study conducted in Northern Chile, 28.7% of female and 17.6% of male participants reported at least one experience of sexual victimization (Ilabaca et al., 2015). In a survey on college students' wellbeing in Santiago, 31% of women and 20% of men reported that they had been made to engage in unwanted sexual activities since the age of 14 (Lehrer et al., 2013a). The third study with fifth-year medical students from Santiago revealed a victimization rate during medical school of 26.4% across both gender groups (separate rates for men and women were not reported; Maida et al., 2003). All three studies only collected victimization reports.

Although the three data sets have yielded important information about the prevalence of sexual victimization in college student samples, the current state of knowledge about sexual aggression in Chile remains limited in several respects. First, no studies are available that obtained reports of sexual aggression perpetration, even though such studies would provide relevant information about the problem of sexual aggression and the relationship between sexual aggression victimization and perpetration. Second, the definitions of sexual aggression varied substantially between the three studies. Maida et al. (2003) applied a broad definition of sexual abuse which included verbal sexual harassment, whereas Ilabaca et al. (2015) and Lehrer et al. (2013b) considered three specific coercive strategies, namely physical force, use of alcohol and/or drugs, and verbal pressure. Third, Lehrer et al. (2013a) and Maida et al. (2003) used only a limited number of items to assess sexual victimization, which may underestimate prevalence rates (Koss, 1993). Fourth, the three studies varied with regard to the time period for which sexual victimization reports were collected, considering only the time in college (Maida et al., 2003), the period since age 14 (Lehrer et al., 2013a), or specifying no timeframe at all (Ilabaca et al., 2015). It is obvious that this heterogeneity undermines the comparability of the findings across studies.

### The current study

The available evidence leaves many questions about sexual aggression among college students in Chile unanswered, in particular referring to sexual aggression perpetration. Therefore, the aim of the current study was to examine the prevalence rates of sexual aggression perpetration and victimization in heterosexual encounters since the legal age of consent in a large sample of college students in Chile. In Chile, the age of consent for heterosexual sexual activities is 14 years, similar to other South American countries, such as Brazil and Colombia. However, the age of consent differs across countries around the world, ranging between 14 and 18 years in the majority of countries.

We used the Sexual Aggression and Victimization Scale (Krahé and Berger, 2013), which is available in Spanish, validated in cross-cultural research, and provides a detailed assessment of sexual aggression victimization and perpetration: It combines three coercive strategies (use or threat of physical force, exploitation of the victim's inability to resist, e.g., due to alcohol or drug use, and use of verbal pressure) with three victim-perpetrator constellations (current or former partners, friends/acquaintances, and strangers). For each combination of coercive strategy and victim-perpetration relationship, four sexual acts are presented (sexual touch, attempted sexual intercourse, completed sexual intercourse, and other sexual activities, such as oral sex). This instrument enabled us to conduct a first comprehensive analysis of sexual aggression victimization and perpetration among college students in Chile. Because our focus was on nonconsensual sexual contacts, we excluded all forms of sexual transgression where lack of consent is not a defining feature, that is sexual contacts with a child under the age of consent and sexual contacts with a person between the ages of 14 and 18 exploiting a relationship of care, where consent is also not an issue as per article 363 of the Chilean penal code.

Based on previous studies showing higher victimization rates for women than for men, we expected that prevalence rates for sexual victimization would be higher for women than for men, and that women would experience more severe forms of sexual victimization. Additionally, we predicted that more women than men would be sole victims, i.e., they would report only victimization but no perpetration (Hypothesis 1). By contrast, we expected that more men than women would report sexual aggression perpetration, that men would report more severe forms of perpetration and that more men than women would be sole perpetrators, i.e., they would report only perpetration but no victimization (Hypothesis 2). Regarding the relationship between victim and perpetrator, we predicted that sexual aggression victimization and perpetration would be more common between (ex-)partners and friends/acquaintances than between strangers (Hypothesis 3). Since past research has shown that alcohol is involved in at least half of all sexually aggressive incidents, we expected a similar pattern in our sample (Hypothesis 4). In addition, we predicted that alcohol consumption would be more common in incidents of sexual aggression victimization and perpetration between strangers than between (ex-)partners and friends/acquaintances (Hypothesis 5). Finally, by assessing both sexual aggression victimization and perpetration, we wanted to examine the prevalence of being both a victim and a perpetrator of sexual aggression.

## Materials and methods

### Participants

The initial sample consisted of *N* = 1310 participants (988 female, 322 male) recruited from five public and private universities located in the Santiago Metropolitan and Valparaíso Region in Chile. From this sample, participants below the age of 18 or 30 years or older were excluded (*n* = 47) because the study was designed to investigate sexual aggression among young adults. A total of 127 participants identified themselves as gay/lesbian (30 women and 39 men) or bisexual (44 women and 14 men). These subgroups were also excluded because heterosexual participants reported a higher age at first sexual intercourse compared to gay/lesbian participants and a lower number of sexual partners compared to bisexual participants. Moreover, gay/lesbian and bisexual participants differed in their sexual behavior, with bisexuals reporting more sexual partners. Therefore, we decided against merging these groups with the large heterosexual sample. We also decided against calculating separate prevalence rates for gay/lesbian and bisexual participants, respectively, as the two groups were too small to yield reliable conclusions. One female participant was excluded because she did not respond to any of the victimization and perpetration items.

The final sample consisted of *N* = 1135 participants (885 female, 250 male), almost all of whom were college students (97.2%) and Chilean nationals (98.6%). The mean age was 22.0 years (*SD* = 2.62, range 18–29 years). Men were significantly older (*M* = 22.4 years, *SD* = 2.68) than women (*M* = 21.8 years, *SD* = 2.59), *t*_(1125)_ = −3.01, *p* < 0.01. Nearly all participants (95.5%) reported non-coital sexual experiences, such as kissing and touching, and 86.3% of the sample had coital experience. The mean age of first sexual intercourse among the participants with coital experience was 17.2 years (*SD* = 2.21). The majority of participants (92.1%) had been in a relationship in the past or were in a relationship at the time of the survey. Men reported more sexual partners (*M* = 2.8, *SD* = 3.10) than did women (*M* = 2.0, *SD* = 2.25), *t*_(277)_ = −3.71, *p* < 0.001.

### Measures

#### Sexual aggression victimization and perpetration

To assess female and male prevalence rates for experiencing and engaging in sexual aggression, the Sexual Aggression and Victimization Scale (SAV-S, Krahé and Berger, 2013) was used. The SAV-S was previously applied in 10 countries, including Spain, and validated by cross-cultural research conducted in several European countries (Krahé et al., 2015, 2016). Similar to the widely used Sexual Experience Survey Short Form (SES-SF, Koss et al., 2007), the SAV-S includes different coercive strategies and specific sexual acts. Unlike the SES, it provides a more detailed picture of the relationship context of sexual assault by assessing, for each specific coercive strategy and sexual act, the relationship between the victim and the perpetrator.

We used a Spanish version of the SAV-S, which had already been used and validated in Spain (Krahé et al., 2015, 2016). Speakers of Chilean Spanish adapted the scale to country-specific language usage. A Chilean Spanish version of the remaining items, originally in German, was created by using the back-translation approach. This involved the translation of the German items into Chilean Spanish by a native speaker of Chile, which were then translated back into German by a native speaker of German.

The items assessing sexual aggression victimization and perpetration are constructed in a parallel way. Three coercive strategies are presented: the use or threat of physical force, the exploitation of the victim's inability to resist (e.g., due to alcohol consumption or drug use), and verbal pressure (e.g., by threatening to end the relationship). After each coercive strategy, three different relationship constellations between the victim and the perpetrator are presented: current or former partner, friend or acquaintance, and stranger. Within each relationship constellation, four sexual acts are presented: sexual touch, attempted sexual intercourse, completed sexual intercourse, and other sexual acts (e.g., oral sex). This cross-classification of coercive strategies, victim-perpetrator relationships, and sexual acts resulted in 36 items each for the victimization and perpetration parts of the SAV-S. Following ethical standards and previous studies (Krahé et al., 2015), participants were asked about victimization first before being prompted to indicate perpetration behavior.

For each item, participants provided frequency ratings on a three-point scale: *never* (0), *once* (1), or *more than once* (>1). Responses were elicited for two time periods: (1) since the age of 14, which represents the legal age of consent for heterosexual sexual contacts in Chile, until 12 months ago, and (2) in the last 12 months. The two time windows correspond to the time periods used by the Sexual Experiences Survey (Koss et al., 2007). The lower age limit of 14 was required to be able to distinguish non-consensual sexual activities from child sexual abuse, where consent is not an issue.

#### Situational drinking behavior

Participants who reported at least one incident of experiencing or engaging in sexual aggression for a particular relationship constellation were asked if alcohol had been consumed in the situation(s) *by themselves* (1), *the other person* (2), *by both of them* (3), or *not at all* (4).

#### Sexual orientation and experience

Participants' sexual orientation was assessed by presenting three options: heterosexual, homosexual, and bisexual. Regarding past sexual behavior, participants were asked to indicate (yes/no) if they had consensual non-coital experiences (e.g., kissing and touching) and had had sexual intercourse. If they reported sexual intercourse, they were asked to indicate their age at first sexual intercourse and the number of partners with whom they had sexual intercourse, separately for steady relationships and casual sexual encounters.

#### Demographic and relationship information

Participants were asked to indicate their sex (male or female), age, nationality, college student status (yes/no), if they had ever been in a relationship, and if they were in a relationship at the time of the study (yes/no).

### Procedure

Approval of the study protocol and all instruments was obtained from the institutional review boards of the authors' universities. The data were collected in an online survey during the second semester of 2015. To recruit participants, information about the study was presented in classes with the approval of the head of department and the instructor, and e-mail addresses of interested students were collected on a voluntary basis to send them the link to the survey. The study was also advertised via institutional e-mail lists, flyers distributed on campuses, and invitations through social media university groups. Participants were required to give active consent on the first page of the questionnaire before being able to proceed to the items.

To account for the possibility that responding to the items might cause distress, a “help button” was provided on each page of the victimization and perpetration questions, which opened a new browser window with information about local counseling centers specializing in sexual violence. This approach follows recommendations of good practice in research on sexual aggression (Krahé and Vanwesenbeeck, 2016). In return for participation, participants were invited to take part in a raffle of gift cards worth $5000 CLP ($7 USD). For this, participants were asked to indicate their e-mail address on a new browser page so that it was stored separately from their answers, ensuing full anonymity.

### Plan of analysis

If participants responded *never* to all victimization items, they were categorized as *non-victims* (coded 0). If they reported at least one incident of sexual victimization, they were categorized as *victim* (coded 1). Perpetration status was coded in the same way. To arrive at a score of lifetime prevalence of sexual aggression victimization and perpetration for each participant, we combined the two time periods.

Because the data were non-normally distributed, we decided to apply non-parametric statistics. Accordingly, for Hypotheses 1 and 2, gender differences in rates for sexual aggression victimization and perpetration and in the sole victimization and perpetration groups were tested through Pearson's chi square test. Since multiple responses to the victimization and perpetration items were possible, differences in the frequency of relationship constellations in sexually aggressive incidents and alcohol consumption predicted in Hypotheses 3 and 5 could not be tested for significance.

## Results

### Prevalence of sexual aggression victimization

Across all coercive strategies, victim-perpetrator constellations, and sexual acts, 51.9% of women and 48.0% of men reported sexual victimization since the age of 14. Prevalence rates of sexual victimization broken down by sex, coercive strategy, and victim-perpetrator relationship are presented in Table 1. The gender difference in the overall rate was non-significant, $\chi_{\left(1,\;\text{N}\;=\;1135\right)}^{2}$ = 1.17, *p* = 0.280, disconfirming Hypothesis 1. Looking at specific combinations of coercive strategy and victim-perpetrator relationship, only one significant gender difference was found: More men (14.6%) than women (8.3%) reported sexual victimization by a stranger through the use of verbal pressure, $\chi_{\left(1,\;\text{N}\;=\;1103\right)}^{2}$ = 8.80, *p* < 0.01. In the Supplementary Material, we provide tables for the prevalence rates of sexual victimization broken down by sex, coercive strategy, victim-perpetrator relationship, and sexual acts, combined for the two time windows (i.e., since the age of 14) and for the separate time periods (Table 1_Suppl. for sexual victimization for both time periods combined/since the age of 14, Table 2_Suppl. for sexual victimization since the age of 14 up to 12 months ago, Table 3_Suppl. for sexual victimization in the last 12 months).

**Table 1:** **Sexual aggression victimization in percent since age 14, broken down by sex, coercive strategy, and victim-perpetrator relationship, *N* = 1135 (nf = 885, nm = 250)**.

**Victim–Perpetrator relationship**	**Coercive strategy**							
	**Use/threat of physical force**		**Exploitation of inability to resist**		**Verbal pressure**		**Total relationship (at least one > = 1 per row)**	
	**Women**	**Men**	**Women**	**Men**	**Women**	**Men**	**Women**	**Men**
(Ex-)Partner	26.3	27.2	18.3	17.3	22.1	17.5	36.0	34.8
Friend/Acquaintance	24.7	24.8	21.0	19.8	12.4	13.8	32.1	32.8
Stranger	17.7	20.0	16.4	16.5	8.3^*^	14.6^*^	23.7	25.2
**Total coercive strategy (at least one > = 1 per column)**	43.0	39.2	34.7	29.0	29.1	26.0	51.9	48.0

Gender difference:

* *p < 0.01. Multiple responses were possible*.

To examine the gender difference in the severity of participants' victimization experiences predicted in Hypothesis 1, we adopted the scoring proposed by Koss et al. (2008). They created a non-redundant six level classification of increasing severity ranging from no victimization to rape: (1) *No victimization* includes all participants who did not report any form of sexual victimization. (2) *Sexual contact* describes sexual touching without penetration (e.g., kissing, fondling) but excludes the more severe forms of sexual victimization. (3) *Attempted coercion* refers to attempted anal, oral, or vaginal penetration using verbal pressure but excludes coercion, attempted, or completed rape. (4) *Coercion* describes verbally pressured anal, oral, or vaginal penetration but excludes attempted or completed rape. (5) *Attempted rape* refers to attempted anal, oral, or vaginal penetration through the use or threat of physical force or the exploitation of the victim's incapacitated state but excludes completed penetration. (6) *Rape* describes anal, oral, or vaginal penetration by using or threatening to use physical force or exploiting the victim's inability to resist. Table 2 presents the prevalence rates of sexual victimization according to this classification for female and male participants.

**Table 2:** **Sexual aggression victimization in percent since age 14 based on the scoring proposed by Koss et al. (2008), *N* = 1135 (nf = 885, nm = 250)**.

	**Sexual victimization**	
	**Women**	**Men**
No victimization	48.1	52.0
Sexual contact	10.7	8.4
Attempted coercion	1.1	0.0
Coercion	3.5	4.4
Attempted rape	5.8	2.8
Rape	30.7	32.4

The findings show that the high overall rates of sexual victimization are due to the high prevalence of completed rape, which was reported by 30.7% of female and 32.4% of male participants. The remaining categories had much lower frequencies. Women and men did not differ in the overall distribution or on any of the individual categories, disconfirming Hypothesis 1.

With respect to the relationship between victimization and perpetration, within the victimized group, 55.0% of men and 70.2% of women were sole victims, that is they only experienced sexual victimization and did not report perpetration, the remaining 45% of men and 29.8% of women in the victimization group also reported some form of perpetration. The gender difference was significant, $\chi_{\left(1,\;\text{N}\;=\;573\right)}^{2}$ = 9.91, *p* < 0.01, confirming Hypothesis 1.

### Prevalence of sexual aggression perpetration

The overall prevalence rates of sexual aggression perpetration since the age of 14 across all coercive strategies, relationship constellations, and sexual acts were 16.5% for women and 26.8% for men. Table 3 shows the prevalence rates for sexual aggression perpetration broken down by sex, coercive strategies, and victim-perpetrator relationship. In line with Hypothesis 2, the gender difference in the overall perpetration rate was significant, $\chi_{\left(1,\;\text{N}\;=\;1121\right)}^{2}$ = 13.40, *p* < 0.001. With respect to gender differences for specific constellations of coercive strategy and relationship, more men than women reported sexual aggression perpetration toward a friend or acquaintance through threatening or using physical force, $\chi_{\left(1,\text{N}=1104\right)}^{2}$ = 8.33, *p* < 0.01, and through exploiting the victim's inability to resist, $\chi_{\left(1,\;\text{N}\;=\;1096\right)}^{2}$ = 8.77, *p* < 0.01. More men than women reported sexual aggression perpetration toward a stranger through exploiting his/her inability to resist, $\chi_{\left(1,\;\text{N}\;=\;1096\right)}^{2}$ = 9.74, *p* < 0.01. In the Supplementary Material, we provide tables for the prevalence rates of sexual aggression perpetration broken down by sex, coercive strategy, victim-perpetrator constellation, and sexual acts, since the age of 14 and for the separate time periods (Table 4_Suppl. for sexual perpetration for both time periods combined/since the age of 14, Table 5_Suppl. for sexual perpetration since the age of 14 up to 12 months ago, Table 6_Suppl. for sexual perpetration in the last 12 months).

**Table 3:** **Sexual aggression perpetration in percent since age 14, broken down by sex, coercive strategy, and victim-perpetrator relationship, *N* = 1121 (nf = 871, nm = 250)**.

	**Coercive strategy**							
	**Use/threat of physical force**		**Exploitation of inability to resist**		**Verbal pressure**		**Total relationship (at least one > = 1 per row)**	
**Victim–Perpetrator relationship**	**Women**	**Men**	**Women**	**Men**	**Women**	**Men**	**Women**	**Men**
(Ex-)Partner	5.0	8.5	4.2	7.3	7.3	10.7	11.7^**^	19.6^**^
Friend/acquaintance	4.0^**^	8.5^**^	4.5^**^	9.4^**^	3.7	5.4	8.3^**^	14.9^**^
Stranger	3.5	4.5	2.5^**^	6.6^**^	1.8	3.7	5.1	7.6
**Total coercive strategy (at least one > = 1 per column)**	8.6	12.9	7.7^*^	13.0^*^	9.1	12.0	16.5^***^	26.8^***^

Gender differences:

* *p < 0.017*,

** *p < 0.01*,

*** *p < 0.001*.

*Multiple responses were possible*.

Comparing sexual aggression victimization and perpetration, prevalence rates were significantly higher for victimization among both men [48.0% victimization vs. 26.8% perpetration, $\chi_{\left(1,\text{N}=250\right)}^{2}=$ 38.97, *p* < 0.001] and women [51.9% victimization vs. 16.5% perpetration, $\chi_{\left(1,\;\text{N}\;=\;871\right)}^{2}$ = 120.43, *p* < 0.001].

To test the gender difference in the severity of sexual assault perpetration proposed in Hypothesis 2, we applied the six-level classification of severity by Koss et al. (2008) to the perpetration reports and categorized all participants according to their most severe form of perpetration reported. The results of this classification are presented in Table 4. For the overall distribution of the six-level scores, a significant gender difference was found, $\chi_{\left(5,\;\text{N}\;=\;1121\right)}^{2}$ = 18.17, *p* < 0.01, confirming Hypothesis 2. At the level of the different categories, significantly more women than men fell into the non-perpetrator category, whereas at the other end of the severity spectrum, significantly more men than women reported rape.

**Table 4:** **Sexual aggression perpetration in percent since age 14 based on the scoring proposed by Koss et al. (2008), *N* = 1121 (nf = 871, nm = 250)**.

	**Sexual aggression perpetration**	
	**Women**	**Men**
No perpetration	83.5^***^	73.2^***^
Sexual contact	5.3	6.4
Attempted coercion	0.6	0.4
Coercion	2.9	3.6
Attempted rape	1.5	3.2
Rape	6.3^***^	13.2^***^

Gender differences:

*** *p < 0.001*.

In the perpetration group, 19.4% of men and 6.3% of women were sole perpetrators, i.e., they reported perpetration but no victimization, whereas the majority of perpetrators (80.6% of men and 93.8% of women) also reported victimization. The gender difference was significant, $\chi_{\left(1,\;\text{N}\;=\;211\right)}^{2}$ = 8.47, *p* < 0.01, supporting Hypothesis 2.

### Victim-perpetrator relationship and coercive strategies

With respect to the relationship between victim and perpetrator, sexual victimization by a current or former partner was reported by 36.0% of women and 34.8% of men. A similar percentage of women (32.1%) and men (32.8%) reported victimization by a friend or acquaintance, and the lowest rates were found for victimization by strangers, reported by 23.7% of women and 25.2% of men. For sexual aggression perpetration, 19.6% of men and 11.7% of women reported sexual aggression perpetration toward a current of former partner, followed by a friend or acquaintance (14.9% of men and 8.3% of women). The lowest prevalence rates were found for sexual aggression perpetration toward a stranger, reported by 7.6% of men and 5.1% of women. These findings confirm Hypothesis 3.

Gender differences within each relationship constellation were tested with a corrected alpha level of *p* < 0.05/3 (critical *p* = 0.017) to account for separate tests for the three victim-perpetrator constellations. For sexual victimization, the gender differences were nonsignificant, with $\chi_{\left(1,\;\text{N}\;=\;1135\right)}^{2}$ = 0.13, *p* = 0.717 for current or former partners, $\chi_{\left(1,\;\text{N}\;=\;1135\right)}^{2}$ = 0.05, *p* = 0.832 for friends/acquaintances, and $\chi_{\left(1,\;\text{N}\;=\;1135\right)}^{2}$ = 0.23, *p* = 0.631 for strangers. Regarding sexual aggression perpetration, more men than women reported sexually aggressive behavior toward a current or former partner, $\chi_{\left(1,\;\text{N}\;=\;1121\right)}^{2}$ = 10.37, *p* < 0.01, and toward a friend or acquaintance, $\chi_{\left(1,\;\text{N}\;=\;1120\right)}^{2}$ = 9.58, *p* < 0.01. No gender difference was found for sexually aggressive behaviors toward strangers, $\chi_{\left(1,\;\text{N}\;=\;1120\right)}^{2}$ = 2.43, *p* = 0.119.

Regarding victimization, the most prevalent coercive strategy was the use or threat of physical force, reported by 43.0% of women and 39.2% of men, followed by exploitation of the inability to resist, reported by 34.7% of women and 29.0% of men, and verbal pressure, reported by 29.1% of women and 26.0% of men. Gender differences were tested across the three coercive strategies using a corrected alpha-level of *p* = 0.05/3. No significant gender differences were found for the three coercive strategies, $\chi_{\left(1,\;\text{N}\;=\;1132\right)}^{2}$ = 1.14, *p* = 0.287 for physical force, $\chi_{\left(1,\;\text{N}\;=\;1121\right)}^{2}$ = 2.80, *p* = 0.095 for exploitation, and $\chi_{\left(1,\;\text{N}\;=\;1109\right)}^{2}$ = 0.89, *p* = 0.346 for verbal pressure.

With respect to sexual aggression perpetration, the prevalence rates for the three coercive strategies (use or threat of physical force, exploitation of the victim's inability to resist, and verbal pressure) were similar, ranging between 12.0 and 13.0% for men and between 7.7 and 9.1% for women. Gender differences within each coercive strategy were examined using a corrected alpha level of *p* < 0.05/3 (critical *p* = 0.017). Paralleling our findings for sexual victimization, men and women did not differ in their use or threat of physical force, $\chi_{\left(1,\;\text{N}\;=\;1109\right)}^{2}$ = 4.14, *p* = 0.042, and verbal pressure, $\chi_{\left(1,\;\text{N}\;=\;1095\right)}^{2}$ = 1.72, *p* = 0.189. By contrast, more men than women reported having exploited the victim's inability to resist, $\chi_{\left(1,\;\text{N}\;=\;1100\right)}^{2}$ = 6.56, *p* < 0.017.

### Alcohol use

Regarding alcohol consumption in incidents of sexual victimization, 63.6% of women and 70.0% of men reported that they, the perpetrator, or both had drunk alcohol in at least one of the reported incidents. With respect to sexual aggression perpetration, about half of the female (48.6%) and male (53.7%) perpetrators indicated that alcohol had been consumed by themselves, the other person, or both, in the situations in which they had shown sexually aggressive behavior. These findings are consistent with Hypothesis 4. The gender differences were not significant, $\chi_{\left(1,\;\text{N}\;=\;579\right)}^{2}$ = 1.70, *p* = 0.192 for victimization and $\chi_{\left(1,\;\text{N}\;=\;209\right)}^{2}$ = 0.48, *p* = 0.488 for perpetration.

As predicted in Hypothesis 5, the use of alcohol was more common in victimization and perpetration incidents involving a stranger (79.5% of women and 84.1% of men who reported victimization by a stranger and 68.2% of women and 89.5% of men who reported having engaged in sexual aggression toward a stranger indicated that alcohol was consumed) than in incidents involving a friend or acquaintance (victims: 62.9% of women and 70.7% of men; perpetrators: 50.0% of women and 62.2% of men) and an (ex-)partner (victims: 40.9% of women and 51.7% of men; perpetrators: 31.3% of women and 40.8% of men). No significant gender differences were found. These findings are consistent with Hypothesis 5 that alcohol is primarily involved in incidents of sexual aggression in which the victim and the perpetrator are strangers.

## Discussion

The purpose of the present study was to provide detailed evidence on the prevalence of sexual aggression victimization and perpetration since the age of consent (i.e., 14 years) in a large sample of college students in Chile. Additionally, we explored the role of alcohol in incidents of victimization and perpetration. By collecting reports of both victimization and perpetration from male and female participants, our study adopted a gender-inclusive approach to the study of sexual aggression (Turchik et al., 2016). The age range of our sample was 18–29 years.

Regarding participants' sexual experience background, almost all participants (95.5%) reported non-coital experiences, such as sexual touching and kissing, and 86.4% of the sample reported coital experience. The latter rate was higher than in past studies with young adults in Chile, which provided estimates between 65 and 71% (Lehrer et al., 2009, 2013b; INJUV, 2013). This difference may be due to the age range of our study starting at 18 years, compared to the study by INJUV (2013), which included participants from the age of 15, but it may also reflect an ongoing liberalization of sexual attitudes and behavior, because the estimates provided by Lehrer et al. (2009, 2013b) were based on a study conducted in 2005. In terms of the onset of coital activities, age at first sexual intercourse in our sample (17.2 years) was similar to figures from past national research and findings from Western countries (Durex, 2005; Barrientos, 2010; Reissing et al., 2012).

Across all coercive strategies, victim-perpetrator relationships, and sexual acts, 51.9% of women and 48.0% of men reported sexual aggression victimization. These rates were higher than previous findings from college student samples in Chile (Maida et al., 2003; Lehrer et al., 2013a; Ilabaca et al., 2015), which may be attributed to the more detailed and behaviorally specific assessment of sexual victimization that permits a better detection of nonconsensual sexual experiences (cf., Koss, 1993). Cross-cultural research from Europe found similar victimization rates for both male and female college students in Greece (55.8% for men and 45.5% for women), for men in Cyprus (49.0%), and for women in the Netherlands (52.2%; Krahé et al., 2015). Regarding the severity of victimization incidents, it is noteworthy that the highest prevalence rates were found for rape rather than for less severe forms, such as sexual contact. This finding indicates that if sexual aggression occurs, it is likely to take the most severe form, namely rape, rather than showing a pattern of higher frequencies for the less severe forms of sexual aggression.

With respect to the perpetration of sexually aggressive behavior, the overall perpetration rates were 26.8% for men and 16.5% for women, constituting the first estimates for college students in Chile. In cross-cultural research, similar prevalence rates were obtained in college students samples from Australia (25.0% of men, 12.7% of women) and Singapore (27.0% of men, 19.3% of women; both countries included in Chan et al., 2008) as well as from Turkey (28.9% of men, 14.2% of women; Schuster et al., 2016). Again, the highest prevalence rates were found for the most severe form of sexual aggression perpetration, namely rape.

Looking at sexual aggression victimization and perpetration in combination, we found victimization rates to be substantially higher than perpetration rates in both gender groups (for women: 51.9% victimization vs. 16.5% perpetration; for men: 48.0% victimization vs. 26.8% perpetration). This discrepancy confirms the substantial difference in self-reports about victim and perpetrator behavior reported by past studies (e.g., Kolivas and Gross, 2007; Krahé and Berger, 2013). It may be related to the fact that perpetration behavior is socially disapproved and the possibility that perpetrators may have multiple victims. The finding that the reporting discrepancy held for both men and women speaks against the explanation that it is due to men's misinterpretation or overestimation of female friendliness as a sexual interest cue (Abbey, 1982; Perilloux et al., 2015). Instead, it is consistent with the alternative explanation that victims have first-hand knowledge of their lack of consent, whereas perpetrators need to infer non-consent from the victim's verbal statements and behavior (Kolivas and Gross, 2007).

Concerning gender differences, our prediction in Hypothesis 1 that more women than men would report sexual victimization, was not supported. Overall, there was no significant difference between the prevalence rates for men and women, and the differentiation of sexual victimization experiences by severity did not show gender differences either. Prevalence rates broken down by coercive strategy and relationship constellation revealed only one significant gender difference, with more men than women reporting sexual victimization through verbal pressure by a stranger. This finding may be related to traditional gender stereotypes which assume that men are always willing to have sex. Hence, if a woman wants to have sexual contact with a male stranger, he may be less willing to express refusal and/or she may be less likely to accept his refusal as genuine.

The absence of gender differences in sexual aggression victimization found in our study is contrary to a large body of Western research which has shown higher victimization rates for women than for men (e.g., Breiding et al., 2014; Cantor et al., 2015). However, the findings are consistent with several recent studies from the cross-cultural literature that also found no or only few gender differences (e.g., Brazil: D'Abreu et al., 2013; U.S.: Hines et al., 2012; Turkey: Schuster et al., 2016; Poland: Tomaszewska and Krahé, 2015) or even higher victimization rates among men compared to women (Krahé et al., 2015), challenging traditional views and stereotypes about sexual aggression victimization. More research is needed to understand social processes, cultural variables, sexuality related cognitions (e.g., sexual scripts, sexual self-esteem) as well as risky sexual behavior which may explain the absence of gender differences in victimization, particularly in the non-Western countries.

Although, we did not find many gender differences in the prevalence of sexual victimization, the psychological impact of sexual victimization may be different for women and men. More research is needed to examine the possibility that women may be more adversely affected by the experience of sexual victimization compared to men, possibly as a result of the imbalance of physical strength. The current body of evidence comparing the psychological consequences of sexual assault for male and female victims is limited and does not provide a conclusive picture. Whereas some studies found more negative effects on women, others found no gender difference, and yet others more adverse effects on men (see Peterson et al., 2011, for a review). Future research is needed to clarify the issue of whether sexual assault victimization has a different impact on female and male victims.

As predicted in Hypothesis 2, more men than women reported the perpetration of sexually aggressive behavior, which is in line with past international evidence (Krahé and Berger, 2013; Williams et al., 2014). For prevalence rates according to the level of severity, men also reported more sexual aggression perpetration than women, except for attempted coercion (0.4% of men and 0.6% of women), but the gender difference was only significant for rape. Additionally, significantly more men (19.4%) than women (6.3%) reported being sole perpetrators, i.e., they only reported perpetration but no victimization. This means that the number of participants who were perpetrators only was very low, and substantially lower than the number of participants who were victims only. Similar results were found by Russell and Oswald (2001, 2002) who showed that 62.9% of men and 80.8% of women in the perpetration group had also been sexually victimized. To explain the overlap of victim and perpetrator roles, longitudinal studies that can assess the temporal sequence of victimization and perpetration are needed.

With respect to victim-perpetrator relationship constellations, sexual aggression victimization and perpetration were more common between (ex-)partners and friends/acquaintances than between strangers, which was in line with Hypothesis 3 and past national and international evidence (e.g., Lehrer et al., 2013a; Tomaszewska and Krahé, 2015; Schuster et al., 2016). This indicates that sexual aggression is more prevalent among persons who know each other than between unknown parties, disconfirming stereotyped views on sexual aggression.

Several cultural factors may have contributed to the high male and female victimization rates. First, although sex education is part of formal education in Chile (Ministerio de Salud Subsecretaría de Salud Pública, 2010), it focuses mainly on biological aspects of reproduction and disregards emotional dimensions, pleasure, sexual diversity, and sexual violence (Macintyre et al., 2015). Other potential sources of information may also be limited or provide a view of sexuality influenced by myths and cultural taboos (Macintyre et al., 2015). Another source of information, in particular for men, is pornography (Macintyre et al., 2015), which is associated with gender-stereotypical beliefs about sexuality and with experiencing and engaging in sexual aggression (Peter and Valkenburg, 2016, for a review). Accordingly, young adults' knowledge about sexuality is limited (Barrientos Delgado and Silva Segovia, 2014), which may compromise their sense of sexual self-determination as well as their respect for the rights of their partners. Second, despite a general liberalization of sexuality (González et al., 2007), which includes the acceptance of premarital sexual intercourse and greater sexual autonomy for women (Barrientos Delgado and Silva Segovia, 2014), double standards for male and female sexuality still prevail. In particular, women who engage in casual sex are often regarded as “whores” or “easy,” while the same does not apply to men (Barrientos Delgado and Silva Segovia, 2014). Thus, this image of women may undermine the respect for women's consent. At the same time, men are expected to be sexually active and experienced, and the social pressure to fulfill this expectation may undermine men's ability and confidence to reject unwanted sexual advances. Third, it is not unusual that young adults in Chile do not have an intimate space where they can engage in sexual intercourse (Barrientos Delgado and Silva Segovia, 2014), in particular those who are economically vulnerable or have more conservative parents. They have to rely on opportunities in which they can use rooms of friends, motels, cars, and public spaces, which may pressure them to have sex when these opportunities arise, potentially undermining respect for mutual consent. Fourth, adherence to the values of the Catholic Church, associated with later sexual initiation and fewer sexual partners (Pedersen, 2014), and the simultaneous exposure to information about very liberal sexual behavior in other parts of the world through globalization convey conflicting messages about sexual relationships. This may contribute to blurred boundaries between consensual sexual activities and sexual aggression.

Another cultural factor may be the high alcohol consumption and binge drinking rates among young adults in Chile (Saldivia and Vizcarra, 2012; Mason-Jones and Cabieses, 2015), given that alcohol is a substantial risk factor for sexual assault. In the present study, alcohol was consumed by one or both parties in at least half of all sexual aggression victimization and perpetration incidents, which is consistent with Hypothesis 4 and past research (Abbey et al., 2004, for a review). It is especially noteworthy that, in line with past research, the use of alcohol was more common between strangers than between (ex-)partners and friends/acquaintances, confirming Hypothesis 5. Alcohol consumption with its impairment of cognitive processes may promote the misinterpretation of another person's behaviors and cues as indicating sexual interest (Abbey et al., 2004), which is more likely to be the case among strangers. Accordingly, alcohol may lower the threshold for using sexual coercion, particularly if there is no emotional connection between perpetrator and victim. However, further culture-sensitive research is needed to confirm these tentative explanations and to understand how young adults' sexuality is shaped by cultural norms and expectations, including alcohol-related beliefs and behaviors. Such research should adopt a qualitative approach to gain a better understanding of the meaning and social construction of sexual aggression from both the victim and the perpetrator perspective (Krahé et al., 2016).

### Strengths and limitations

We believe that our study has several strengths. We were able to recruit a large sample of students from different public and private universities located in the Santiago Metropolitan and Valparaíso Region, where about half of the Chilean population lives (Comité Nacional de Estadísticas Vitales, 2015). Our study collected information about sexual aggression victimization and perpetration from both men and women, which means we were able to provide insights in the relationship between being a victim and engaging in sexually aggressive behavior. Additionally, the assessment of coercive strategies, relationship constellations between victim and perpetrator, and sexual acts yielded a detailed picture of sexual aggression victimization and perpetration in a country for which little previous evidence is available.

At the same time, several limitations have to be noted. The first is that we used a convenience sample, and future research is needed to determine whether similar prevalence rates are obtained in other samples of college students and young adults more generally. This limitation notwithstanding, it has been shown that convenience samples may yield valid conclusions (Straus, 2009). A second limitation was that women were overrepresented in the sample, which should be kept in mind when interpreting the male prevalence rates. Third, our initial sample included persons who described their sexual orientation as gay/lesbian or bisexual, however their number was too small to facilitate reliable analyses of prevalence rates. More research is needed both in Chile and internationally about the prevalence of sexual aggression victimization and perpetration among gay, lesbian, and bisexual persons since studies suggest that they may be a vulnerable group (see Rothman et al., 2011, for a review). Fourth, in assessing sexual aggression our focus was on nonconsensual sexual contacts. Therefore, we excluded all forms of sexual transgression where lack of consent is not a defining feature, i.e., childhood sexual abuse by any perpetrator under the age of 14 and sexual contacts by family members, which are based on the exploitation of a relationship of care. To cover all facets of sexual aggression, future research should consider to include family members and other perpetrators exploiting a position of trust or authority.

Despite these limitations, the current study provides new data about the prevalence of sexual aggression among college students in Chile, addressing the knowledge gap about victimization and, in particular, perpetration in this country. Consistent with studies from the international literature, sexual aggression was established to be a serious problem among college students, affecting a substantial proportion of women and men. The findings highlight the need to put sexual aggression on the policy agenda in Chile, documenting the need to develop and implement prevention programs, including the coverage of sexual aggression in formal sex education curricula. Furthermore, our findings contribute to the international data base on sexual aggression and provide a starting point for considering cultural variables that may allow a better understanding of young adults' sexuality and sexual aggression in different parts of the world.

## Author contributions

IS designed the study, collected, analyzed and interpreted the data, and wrote the article. BK provided input and supervision to the design of the study, analyses and interpretation of the data, and writing up the article. PI and JM helped with the process of obtaining ethics approval and with the data collection. All authors critically revised the manuscript, approved this version, and agreed to be accountable for all aspects of this work and its integrity.

## Funding

The study was supported by a grant from the German Academic Scholarship Foundation (Studienstiftung des Deutschen Volkes) to the first author. We also gratefully acknowledge the support of the Deutsche Forschungsgemeinschaft (German Research Foundation) and the Open Access Publishing Fund of the University of Potsdam.

### Conflict of interest statement

The authors declare that the research was conducted in the absence of any commercial or financial relationships that could be construed as a potential conflict of interest.
