# Supplementary material for: Sexual Aggression Victimization and Perpetration among Male and Female College Students in Chile
Source: Front Psychol. 2016 Sep 21;7:1354. doi: 10.3389/fpsyg.2016.01354 (PMC5030277; doi:10.3389/fpsyg.2016.01354)
Supplement: Supplementary file 1 [file Table1.DOCX]

***Supplementary Material***

**Sexual Aggression Victimization and Perpetration Among Male and Female College Students in Chile**

**Isabell Schuster^*^, Barbara Krahé, Paola Ilabaca Baeza and José Antonio Muñoz Reyes**

**Correspondence:** Isabell Schuster: [isschust@uni-potsdam.de](mailto:isschust@uni-potsdam.de)

**Prevalence of Specific Forms of Sexual Aggression *Victimization and Perpetration***

Table 1_Suppl.

*Sexual Victimization in Percent, Broken Down by Sex, Coercive Strategy, Victim – Perpetrator Relationship and Type of Sexual Activity for Both Time Periods Combined (= Since Age 14), N = 1,135 (n_f_ = 885, n_m_ = 250)*

|  |  |  | |  |  |  |  |  | | Coercive Strategy | | | | | | | | | | | | | |
| --- | --- | --- | --- | --- | --- | --- | --- | --- | --- | --- | --- | --- | --- | --- | --- | --- | --- | --- | --- | --- | --- | --- | --- |
|  | | |  | |  | Use/Threat of Physical Force | | | | |  | Exploitation  of the Inability to Resist | | | |  | Verbal Pressure | | |  | Overall  (at least one  ≥ 1 per row) | | |
| Victim-Perpetrator  Relationship | | | Sexual Activity | |  | Women | |  | Men | |  | Women | |  | Men |  | Women |  | Men |  | Women |  | Men |
| (Ex-)Partner | | | Touching | |  | 21.0 | |  | 22.0 | |  | | 15.5 |  | 16.5 |  | 18.1 |  | 15.4 |  | 30.3 |  | 31.6 |
|  | | | Attempted sex. inter. | |  | 16.3 | |  | 21.2 | |  | | 12.3 |  | 13.8 |  | 16.3 |  | 13.4 |  | 25.0 |  | 27.2 |
|  | | | Completed sex. inter. | |  | 12.3** | |  | 19.2** | |  | | 9.9 |  | 10.9 |  | 13.4 |  | 12.2 |  | 20.8 |  | 26.0 |
|  | | | Other (e.g., oral sex) | |  | 13.8 | |  | 19.6 | |  | | 9.4 |  | 13.0 |  | 13.3 |  | 13.0 |  | 21.2 |  | 26.0 |
| Total (Ex-)Partner | | | | |  | *26.3* | |  | *27.2* | |  | | *18.3* |  | *17.3* |  | *22.1* |  | *17.5* |  | *36.0* |  | *34.8* |
| Friend/Acquaintance | | | Touching | |  | 21.9 | |  | 22.4 | |  | | 18.9 |  | 19.0 |  | 11.0 |  | 13.0 |  | 29.6 |  | 31.2 |
|  | | | Attempted sex. inter. | |  | 13.0 | |  | 16.9 | |  | | 11.1 |  | 14.9 |  | 7.6 |  | 10.2 |  | 18.4 |  | 22.4 |
|  | | | Completed sex. inter. | |  | 8.7** | |  | 15.6** | |  | | 7.9 |  | 12.9 |  | 4.5** |  | 9.3** |  | 13.3 |  | 19.6 |
|  | | | Other (e.g., oral sex) | |  | 8.7*** | |  | 17.6*** | |  | | 7.9 |  | 12.9 |  | 4.9** |  | 10.2** |  | 13.1*** |  | 22.8*** |
| Total Friend/Acquaintance | | | | |  | *24.7* | |  | *24.8* | |  | | *21.0* |  | *19.8* |  | *12.4* |  | *13.8* |  | *32.1* |  | *32.8* |
| Stranger | | | Touching | |  | 16.4 | |  | 20.0 | |  | | 15.5 |  | 15.7 |  | 7.8 |  | 13.0 |  | 22.8 |  | 24.0 |
|  | | | Attempted sex. inter. | |  | 7.1** | |  | 13.7** | |  | | 7.3 |  | 11.7 |  | 3.4*** |  | 9.8*** |  | 11.2 |  | 17.2 |
|  | | | Completed sex. inter. | |  | 4.2*** | |  | 11.6*** | |  | | 4.4*** |  | 11.7*** |  | 2.0*** |  | 7.3*** |  | 6.9*** |  | 15.2*** |
|  | | | Other (e.g., oral sex) | |  | 4.2*** | |  | 12.4*** | |  | | 4.5*** |  | 11.3*** |  | 2.9*** |  | 10.2*** |  | 7.5*** |  | 16.4*** |
| Total Stranger | | | | |  | *17.7* | |  | *20.0* | |  | | *16.4* |  | *16.5* |  | *8.3*** |  | *14.6*** |  | *23.7* |  | *25.2* |
| Total Coercive Strategy | | | | |  | *43.0* | |  | *39.2* | |  | | *34.7* |  | *29.0* |  | *29.1* |  | *26.0* |  | *51.9* |  | *48.0* |

*Note*. ** *p* < .01, *** *p* < .001. Sex. inter. = Sexual intercourse. Multiple responses were possible.

Table 2_Suppl.

*Sexual Victimization in Percent, Broken Down by Sex, Coercive Strategy, Victim – Perpetrator Relationship and Type of Sexual Activity since Age 14 up to 12 Months ago*, *N* = 1,089 (*n*_f_ = 853, *n*_m_ = 236)

|  |  |  | |  |  |  |  |  | | Coercive Strategy | | | | | | | | | | | | |
| --- | --- | --- | --- | --- | --- | --- | --- | --- | --- | --- | --- | --- | --- | --- | --- | --- | --- | --- | --- | --- | --- | --- |
|  | | |  | |  | Use/Threat of Physical Force | | | | |  | Exploitation  of Inability to Resist | | |  | Verbal Pressure | | |  | Overall  (at least one  >=1 per row) | | |
| Victim – Perpetrator  Relationship | | | Sexual activity | |  | Women | |  | Men | |  | Women |  | Men |  | Women |  | Men |  | Women |  | Men |
| (Ex-)Partner | | | Touching | |  | 15.0 | |  | 14.5 | |  | 10.6 |  | 10.2 |  | 13.0 |  | 8.4 |  | 20.9 |  | 20.5 |
|  | | | Attempted sex. inter. | |  | 11.5 | |  | 12.6 | |  | 8.6 |  | 8.0 |  | 11.7 |  | 7.5 |  | 17.0 |  | 16.2 |
|  | | | Completed sex. inter. | |  | 8.4 | |  | 10.4 | |  | 7.0 |  | 6.7 |  | 8.6 |  | 6.3 |  | 13.1 |  | 15.4 |
|  | | | Other (e.g., oral sex) | |  | 8.3** | |  | 14.4** | |  | 6.0 |  | 7.6 |  | 8.3 |  | 7.1 |  | 12.5 |  | 17.1 |
| Total (Ex-)Partner | | | | |  | *18.9* | |  | *19.3* | |  | *12.6* |  | *10.1* |  | *15.2* |  | *9.3* |  | *24.9* |  | *23.5* |
| Friend/Acquaintance | | | Touching | |  | 16.4 | |  | 13.8 | |  | 14.2 |  | 10.7 |  | 8.2 |  | 9.2 |  | 22.5 |  | 20.1 |
|  | | | Attempted sex. inter. | |  | 8.6 | |  | 10.4 | |  | 7.6 |  | 8.0 |  | 5.1 |  | 6.6 |  | 12.5 |  | 13.7 |
|  | | | Completed sex. inter. | |  | 5.5 | |  | 8.6 | |  | 5.2 |  | 7.1 |  | 2.7 |  | 6.1 |  | 8.4 |  | 11.5 |
|  | | | Other (e.g., oral sex) | |  | 5.3** | |  | 10.3** | |  | 5.4 |  | 7.6 |  | 3.1 |  | 6.6 |  | 8.4 |  | 13.7 |
| Total Friend/Acquaintance | | | | |  | *18.0* | |  | *15.6* | |  | *15.4* |  | *11.5* |  | *8.9* |  | *9.6* |  | *24.4* |  | *21.4* |
| Stranger | | | Touching | |  | 10.3 | |  | 9.4 | |  | 10.4 |  | 9.3 |  | 5.1 |  | 7.9 |  | 15.8 |  | 14.0 |
|  | | | Attempted sex. inter. | |  | 4.2 | |  | 6.3 | |  | 4.8 |  | 6.7 |  | 2.4** |  | 6.1** |  | 7.2 |  | 9.0 |
|  | | | Completed sex. inter. | |  | 2.6 | |  | 5.4 | |  | 2.6** |  | 6.2** |  | 1.1*** |  | 4.9*** |  | 4.0 |  | 8.1 |
|  | | | Other (e.g., oral sex) | |  | 2.2** | |  | 6.7** | |  | 2.3** |  | 6.7** |  | 1.4*** |  | 6.6*** |  | 3.8*** |  | 9.8*** |
| Total Stranger | | | | |  | *11.2* | |  | *10.6* | |  | *11.1* |  | *9.3* |  | *5.3* |  | *8.7* |  | *16.6* |  | *15.3* |
| Total Coercive Strategy | | | | |  | *31.5* | |  | *26.8* | |  | *26.0*** |  | *17.5*** |  | *19.9* |  | *15.3* |  | *38.6* |  | *33.1* |

*Note*. ** *p* < .01, *** *p* < .001. Sex. inter. = Sexual intercourse. Multiple responses were possible.

Table 3_Suppl.

*Sexual Victimization in Percent, Broken Down by Sex, Coercive Strategy, Victim – Perpetrator Relationship and Type of Sexual Activity in the Last 12 Months*, *N* = 1,053 (*n*_f_ = 817, *n*_m_ = 236)

|  |  |  | |  |  |  |  |  | | Coercive Strategy | | | | | | | | | | | | |
| --- | --- | --- | --- | --- | --- | --- | --- | --- | --- | --- | --- | --- | --- | --- | --- | --- | --- | --- | --- | --- | --- | --- |
|  | | |  | |  | Use/Threat of Physical Force | | | | |  | Exploitation  of Inability to Resist | | |  | Verbal Pressure | | |  | Overall  (at least one  >=1 per row) | | |
| Victim – Perpetrator  Relationship | | | Sexual activity | |  | Women | |  | Men | |  | Women |  | Men |  | Women |  | Men |  | Women |  | Men |
| (Ex-)Partner | | | Touching | |  | 11.6 | |  | 16.5 | |  | 9.5 |  | 13.4 |  | 9.9 |  | 13.6 |  | 18.5 |  | 25.0 |
|  | | | Attempted sex. inter. | |  | 9.4** | |  | 16.6** | |  | 7.0 |  | 10.8 |  | 8.1 |  | 11.4 |  | 15.5 |  | 21.7 |
|  | | | Completed sex. inter. | |  | 7.1*** | |  | 16.6*** | |  | 5.6 |  | 9.4 |  | 7.8 |  | 10.5 |  | 13.4** |  | 21.8** |
|  | | | Other (e.g., oral sex) | |  | 8.6** | |  | 14.5** | |  | 5.9 |  | 10.9 |  | 7.8 |  | 11.4 |  | 13.7** |  | 22.1** |
| Total (Ex-)Partner | | | | |  | *15.7* | |  | *22.1* | |  | *10.9* |  | *15.2* |  | *12.5* |  | *16.4* |  | *23.6* |  | *29.2* |
| Friend/Acquaintance | | | Touching | |  | 11.6** | |  | 18.3** | |  | 9.7** |  | 16.8** |  | 6.0 |  | 10.6 |  | 15.7*** |  | 26.1*** |
|  | | | Attempted sex. inter. | |  | 7.9** | |  | 14.5** | |  | 6.2*** |  | 14.1*** |  | 4.1** |  | 9.3** |  | 10.6*** |  | 20.9*** |
|  | | | Completed sex. inter. | |  | 5.5*** | |  | 13.5*** | |  | 4.6** |  | 10.9** |  | 2.9*** |  | 8.3*** |  | 8.1*** |  | 17.8*** |
|  | | | Other (e.g., oral sex) | |  | 5.8*** | |  | 16.3*** | |  | 4.5*** |  | 12.8*** |  | 2.9*** |  | 8.8*** |  | 8.2*** |  | 21.7*** |
| Total Friend/Acquaintance | | | | |  | *13.7*** | |  | *21.0*** | |  | *11.1* |  | *17.6* |  | *6.9* |  | *12.0* |  | *17.8**** |  | *28.7**** |
| Stranger | | | Touching | |  | 10.9 | |  | 16.9 | |  | 8.9 |  | 13.9 |  | 4.9** |  | 11.1** |  | 13.7 |  | 20.4 |
|  | | | Attempted sex. inter. | |  | 4.7*** | |  | 11.7*** | |  | 4.2*** |  | 10.6*** |  | 2.1*** |  | 7.8*** |  | 6.8*** |  | 15.2*** |
|  | | | Completed sex. inter. | |  | 2.9*** | |  | 9.9*** | |  | 2.7*** |  | 10.6*** |  | 1.3*** |  | 5.6*** |  | 4.5*** |  | 13.0*** |
|  | | | Other (e.g., oral sex) | |  | 3.2*** | |  | 10.9*** | |  | 3.2*** |  | 9.7*** |  | 2.1*** |  | 8.8*** |  | 5.3*** |  | 15.2*** |
| Total Stranger | | | | |  | *11.6* | |  | *17.3* | |  | *9.4*** |  | *15.7*** |  | *5.3**** |  | *13.4**** |  | *14.1*** |  | *22.2*** |
| Total Coercive Strategy | | | | |  | *26.6* | |  | *33.6* | |  | *19.2* |  | *25.2* |  | *16.9* |  | *24.0* |  | *33.4** |  | *41.5** |

*Note*. * *p* < .05, ** *p* < .01, *** *p* < .001. Sex. inter. = Sexual intercourse. Multiple responses were possible.

Table 4_Suppl.

*Sexual Perpetration in Percent, Broken Down by Sex, Coercive Strategy, Victim – Perpetrator Relationship and Type of Sexual Activity for Both Time Periods Combined (= Since Age 14), N = 1,121 (n_f_ = 871, n_m_ = 250)*

|  |  |  | |  |  |  |  |  | | Coercive Strategy | | | | | | | | | | | | |
| --- | --- | --- | --- | --- | --- | --- | --- | --- | --- | --- | --- | --- | --- | --- | --- | --- | --- | --- | --- | --- | --- | --- |
|  | | |  | |  | Use/Threat of Physical Force | | | | |  | Exploitation  of the Inability to Resist | | |  | Verbal Pressure | | |  | Overall  (at least one  ≥ 1 per row) | | |
| Victim-Perpetrator  Relationship | | | Sexual Activity | |  | Women | |  | Men | |  | Women |  | Men |  | Women |  | Men |  | Women |  | Men |
| (Ex-)Partner | | | Touching | |  | 4.7 | |  | 7.7 | |  | 3.6 |  | 5.7 |  | 5.9 |  | 8.3 |  | 10.1** |  | 16.1** |
|  | | | Attempted sex. inter. | |  | 2.9 | |  | 4.5 | |  | 2.1 |  | 4.9 |  | 3.5 |  | 6.6 |  | 6.2 |  | 10.4 |
|  | | | Completed sex. inter. | |  | 2.1 | |  | 3.2 | |  | 1.9** |  | 5.7** |  | 2.4** |  | 6.6** |  | 5.1** |  | 10.0** |
|  | | | Other (e.g., oral sex) | |  | 1.6 | |  | 3.2 | |  | 1.6 |  | 3.7 |  | 2.6 |  | 5.0 |  | 4.5 |  | 7.6 |
| Total (Ex-)Partner | | | | |  | *5.0* | |  | *8.5* | |  | *4.2* |  | *7.3* |  | *7.3* |  | *10.7* |  | *11.7*** |  | *19.6*** |
| Friend/Acquaintance | | | Touching | |  | 3.7** | |  | 8.5** | |  | 4.0 |  | 7.3 |  | 3.1 |  | 4.6 |  | 7.6** |  | 12.9** |
|  | | | Attempted sex. inter. | |  | 2.0 | |  | 4.9 | |  | 1.9** |  | 4.9** |  | 1.3 |  | 3.3 |  | 3.6*** |  | 9.2*** |
|  | | | Completed sex. inter. | |  | 1.5 | |  | 2.4 | |  | 1.6** |  | 4.9** |  | 1.4 |  | 2.9 |  | 3.2** |  | 7.2** |
|  | | | Other (e.g., oral sex) | |  | 1.3** | |  | 4.9** | |  | 1.8** |  | 5.0** |  | 1.2** |  | 3.7** |  | 3.1** |  | 8.0** |
| Total Friend/Acquaintance | | | | |  | *4.0*** | |  | *8.5*** | |  | *4.5*** |  | *9.4*** |  | *3.7* |  | *5.4* |  | *8.3*** |  | *14.9*** |
| Stranger | | | Touching | |  | 3.4 | |  | 4.5 | |  | 1.9** |  | 5.7** |  | 1.5 |  | 3.7 |  | 4.6 |  | 7.2 |
|  | | | Attempted sex. inter. | |  | 1.6 | |  | 2.8 | |  | 1.2*** |  | 4.9*** |  | 0.8 |  | 2.5 |  | 2.3** |  | 6.0** |
|  | | | Completed sex. inter. | |  | 0.7 | |  | 2.4 | |  | 0.9** |  | 3.3** |  | 0.5 |  | 1.7 |  | 1.6 |  | 4.0 |
|  | | | Other (e.g., oral sex) | |  | 0.7** | |  | 3.2** | |  | 0.6*** |  | 4.5*** |  | 0.5** |  | 2.5** |  | 1.5** |  | 5.2** |
| Total Stranger | | | | |  | *3.5* | |  | *4.5* | |  | *2.5*** |  | *6.6*** |  | *1.8* |  | *3.7* |  | *5.1* |  | *7.6* |
| Total Coercive Strategy | | | | |  | *8.6* | |  | *12.9* | |  | *7.7** |  | *13.0** |  | *9.1* |  | *12.0* |  | *16.5**** |  | *26.8**** |

*Note*. * *p* < .05, ** *p* < .01, *** *p* < .001. Sex. inter. = Sexual intercourse. Multiple responses were possible.

Table 5_Suppl.

*Sexual Perpetration in Percent, Broken Down by Sex, Coercive Strategy, Victim – Perpetrator Relationship and Type of Sexual Activity since Age 14 up to 12 Months ago*, *N* = 1,071 (*n*_f_ = 834, *n*_m_ = 237)

|  |  |  | |  |  |  |  |  | | Coercive Strategy | | | | | | | | | | | | |
| --- | --- | --- | --- | --- | --- | --- | --- | --- | --- | --- | --- | --- | --- | --- | --- | --- | --- | --- | --- | --- | --- | --- |
|  | | |  | |  | Use/Threat of Physical Force | | | | |  | Exploitation | | |  | Verbal Pressure | | |  | Overall  (at least one  >=1 per row) | | |
| Victim – Perpetrator  Relationship | | | Sexual activity | |  | Women | |  | Men | |  | Women |  | Men |  | Women |  | Men |  | Women |  | Men |
| (Ex-)Partner | | | Touching | |  | 2.7 | |  | 4.8 | |  | 1.2** |  | 3.9** |  | 3.1 |  | 3.5 |  | 5.7 |  | 8.5 |
|  | | | Attempted sex. inter. | |  | 1.7 | |  | 2.6 | |  | 0.9** |  | 3.5** |  | 2.1 |  | 1.8 |  | 3.6 |  | 4.7 |
|  | | | Completed sex. inter. | |  | 1.1 | |  | 1.7 | |  | 0.7** |  | 3.5** |  | 1.5 |  | 1.8 |  | 2.8 |  | 4.7 |
|  | | | Other (e.g. oral sex) | |  | 0.7 | |  | 2.6 | |  | 0.7 |  | 2.2 |  | 1.4 |  | 1.8 |  | 2.3 |  | 4.3 |
| Total (Ex-)Partner | | | | |  | *3.1* | |  | *5.2* | |  | *1.6*** |  | *4.8*** |  | *3.8* |  | *4.8* |  | *6.7* |  | *10.6* |
| Friend/Acquaintance | | | Touching | |  | 2.2 | |  | 5.2 | |  | 2.2 |  | 4.4 |  | 1.5 |  | 2.7 |  | 4.3 |  | 7.6 |
|  | | | Attempted sex. Inter. | |  | 1.0 | |  | 2.6 | |  | 1.1 |  | 3.1 |  | 0.7 |  | 1.3 |  | 1.8 |  | 4.7 |
|  | | | Completed sex. Inter. | |  | 0.7 | |  | 1.3 | |  | 0.9 |  | 3.1 |  | 0.6 |  | 0.9 |  | 1.7 |  | 3.8 |
|  | | | Other (e.g. oral sex) | |  | 0.6** | |  | 3.0** | |  | 0.9 |  | 3.1 |  | 0.5 |  | 1.8 |  | 1.3** |  | 4.7** |
| Total Friend/Acquaintance | | | | |  | *2.3* | |  | *5.6* | |  | *2.7* |  | *6.1* |  | *2.1* |  | *3.6* |  | *5.0** |  | *9.3** |
| Stranger | | | Touching | |  | 2.1 | |  | 2.2 | |  | 1.4 |  | 3.5 |  | 0.5 |  | 2.2 |  | 2.8 |  | 4.7 |
|  | | | Attempted sex. inter. | |  | 1.1 | |  | 1.3 | |  | 0.5** |  | 3.1** |  | 0.6 |  | 1.3 |  | 1.3 |  | 3.4 |
|  | | | Completed sex. inter. | |  | 0.2 | |  | 0.9 | |  | 0.6 |  | 1.8 |  | 0.4 |  | 0.9 |  | 1.0 |  | 2.1 |
|  | | | Other (e.g. oral sex) | |  | 0.4 | |  | 1.8 | |  | 0.2*** |  | 2.6*** |  | 0.2** |  | 1.8** |  | 0.7** |  | 3.0** |
| Total Stranger | | | | |  | *2.2* | |  | *2.2* | |  | *1.7* |  | *3.9* |  | *0.7* |  | *2.2* |  | *3.1* |  | *4.7* |
| Total Coercive Strategy | | | | |  | *5.6* | |  | *8.2* | |  | *4.4** |  | *8.6** |  | *5.0* |  | *5.7* |  | *10.4** |  | *15.2** |

*Note*. * *p* < .05, ** *p* < .01, *** *p* < .001. Sex. inter. = Sexual intercourse. Multiple responses were possible.

Table 6_Suppl.

*Sexual Perpetration in Percent, Broken Down by Sex, Coercive Strategy, Victim – Perpetrator Relationship and Type of Sexual Activity in the Last 12 Months*, *N* = 1,032 (*n*_f_ = 800, *n*_m_ = 232)

|  |  |  | |  |  |  |  | |  | | Coercive Strategy | | | | | | | | | | | | |
| --- | --- | --- | --- | --- | --- | --- | --- | --- | --- | --- | --- | --- | --- | --- | --- | --- | --- | --- | --- | --- | --- | --- | --- |
|  | | |  | |  | Use/Threat of Physical Force | | | | | |  | Exploitation | | |  | Verbal Pressure | | |  | Overall  (at least one  >=1 per row) | | |
| Victim – Perpetrator  Relationship | | | Sexual activity | |  | Women | |  | | Men | |  | Women |  | Men |  | Women |  | Men |  | Women |  | Men |
| (Ex-)Partner | | | Touching | |  | 3.2 | |  | | 4.5 | |  | 2.9 |  | 4.5 |  | 4.0 |  | 6.4 |  | 7.0 |  | 10.9 |
|  | | | Attempted sex. inter. | |  | 1.7 | |  | | 2.7 | |  | 1.4 |  | 4.1 |  | 2.1** |  | 6.0** |  | 3.6** |  | 8.7** |
|  | | | Completed sex. inter. | |  | 1.3 | |  | | 1.8 | |  | 1.3** |  | 4.6** |  | 1.2*** |  | 5.5*** |  | 2.9** |  | 7.8** |
|  | | | Other (e.g. oral sex) | |  | 1.3 | |  | | 1.3 | |  | 1.0** |  | 3.7** |  | 1.4** |  | 4.6** |  | 2.8 |  | 6.1 |
| Total (Ex-)Partner | | | | |  | *3.2* | |  | | *4.9* | |  | *3.2* |  | *5.9* |  | *5.1* |  | *8.3* |  | *8.2** |  | *13.5** |
| Friend/Acquaintance | | | Touching | |  | 2.5** | |  | | 7.2** | |  | 2.6 |  | 6.0 |  | 2.5 |  | 3.2 |  | 5.2** |  | 10.4** |
|  | | | Attempted sex. inter. | |  | 1.6 | |  | | 4.1 | |  | 1.4 |  | 3.7 |  | 1.1 |  | 2.8 |  | 2.9** |  | 7.4** |
|  | | | Completed sex. inter. | |  | 1.2 | |  | | 2.7 | |  | 1.3 |  | 3.7 |  | 1.2 |  | 2.8 |  | 2.5** |  | 6.1** |
|  | | | Other (e.g. oral sex) | |  | 1.2 | |  | | 3.6 | |  | 1.2** |  | 4.2** |  | 1.2 |  | 3.2 |  | 2.8** |  | 7.0** |
| Total Friend/Acquaintance | | | | |  | *2.6*** | |  | | *7.2*** | |  | *2.9*** |  | *6.9*** |  | *2.9* |  | *4.1* |  | *5.8*** |  | *11.7*** |
| Stranger | | | Touching | |  | 2.3 | |  | | 4.1 | |  | 1.2** |  | 4.6** |  | 1.4 |  | 2.7 |  | 3.2 |  | 5.7 |
|  | | | Attempted sex. inter. | |  | 1.1 | |  | | 2.7 | |  | 1.1** |  | 4.2** |  | 0.5 |  | 1.8 |  | 1.9** |  | 5.3** |
|  | | | Completed sex. inter. | |  | 0.7 | |  | | 2.7 | |  | 0.5** |  | 3.3** |  | 0.3 |  | 1.4 |  | 1.1** |  | 3.9** |
|  | | | Other (e.g. oral sex) | |  | 0.4** | |  | | 2.7** | |  | 0.4*** |  | 4.2*** |  | 0.4 |  | 1.8 |  | 1.0*** |  | 4.8*** |
| Total Stranger | | | | |  | *2.4* | |  | | *4.1* | |  | *1.5**** |  | *5.6**** |  | *1.7* |  | *2.7* |  | *3.4* |  | *6.1* |
| Total Coercive Strategy | | | | |  | *5.5* | |  | | *9.8* | |  | *5.6* |  | *10.0* |  | *6.4* |  | *9.5* |  | *11.0**** |  | *19.8**** |

*Note*. * *p* < .05, ** *p* < .01, *** *p* < .001. Sex. inter. = Sexual intercourse. Multiple responses were possible.
